# Early Career Reviewer Hub: Paving the way for the next generation of peer reviewers

**DOI:** 10.1002/2211-5463.70335

**Published:** 2026-09-04

**Authors:** Sara Fuentes, Miguel A. De la Rosa

**Affiliations:** ^1^ FEBS Open Bio Editorial Office Cambridge UK; ^2^ Institute for Chemical Research (IIQ), Scientific Research Centre Isla de la Cartuja (cicCartuja), Universidad de Sevilla‐CSIC Spain

## Abstract

*FEBS Open Bio* is pleased to announce the launch of the Early Career Reviewer Hub, a new initiative designed to provide professional training and first‐hand experience to Early Career Researchers (ECRs) interested in taking part in the peer‐review process. We hope that, through this new programme and our focus on Research Protocols, we will continue to support and contribute to the professional development of early career molecular life scientists.

**Abbreviation**
ECR: Early Career Researcher

It is difficult to imagine scholarly research without the safeguarding provided by the peer‐review process. However, for most of humankind's existence, knowledge was largely passed down from individuals through personal communication, letters, scholarly society presentations and books, with limited public scrutiny. In 1665, The Royal Society of London began publishing the *Philosophical Transactions of the Royal Society*, which marked the beginning of scientific journals [1]. This periodical published original research in all areas of natural philosophy, and although inclusion in the journal was mainly driven by the editor's opinion, it arguably constitutes the earliest recorded form of peer review.

For approximately the next 300 years, the peer‐review process relied exclusively on the opinion of a few distinguished experts who decided on the merit and interest of the work based on their sole judgement and expertise. The shortcomings of this form of peer review are obvious, ranging from personal prejudices to the lack of cross‐checking. This type of peer review also heavily favoured incremental scientific advances that built on established theories and more groundbreaking ideas were more easily ignored or ridiculed. Perhaps nothing illustrates this better than the backlash faced by Ignaz Semmelweis when he presented his work on antiseptic measures to avert childbed fever or MMR. Established medical journals such as the *Gazette médicale de Paris* concluded that despite the data, the fact that deaths by MMR fell after the implementation of chlorine hand disinfection was not sufficient to conclude that the measure caused the fall, as this ‘could be due to completely different circumstances’ [2].

By the second half of the 20th century, the number of published articles increased drastically (Fig. 1A). This increase was also accompanied by a greater diversity in research topics, as shown by the higher density of word clusters formed by the terms present in the title and abstracts of *The FEBS Journal* articles published in 2025 (Fig. 1B) compared with those from articles published in 1975 (Fig. 1C). With the surge in the number of scientific manuscripts and the diversification of scientific research, it became increasingly difficult for editors to manage the workload and make informed decisions without external input. However, it was not until the 1970s that external peer review became more systematic and standardised. For much of the 20th century, many authors agreed that refereeing felt erratic, with editors and referees often taking more notice of the authors' identities and their affiliations than the content of the research [3].

**Fig. 1:**
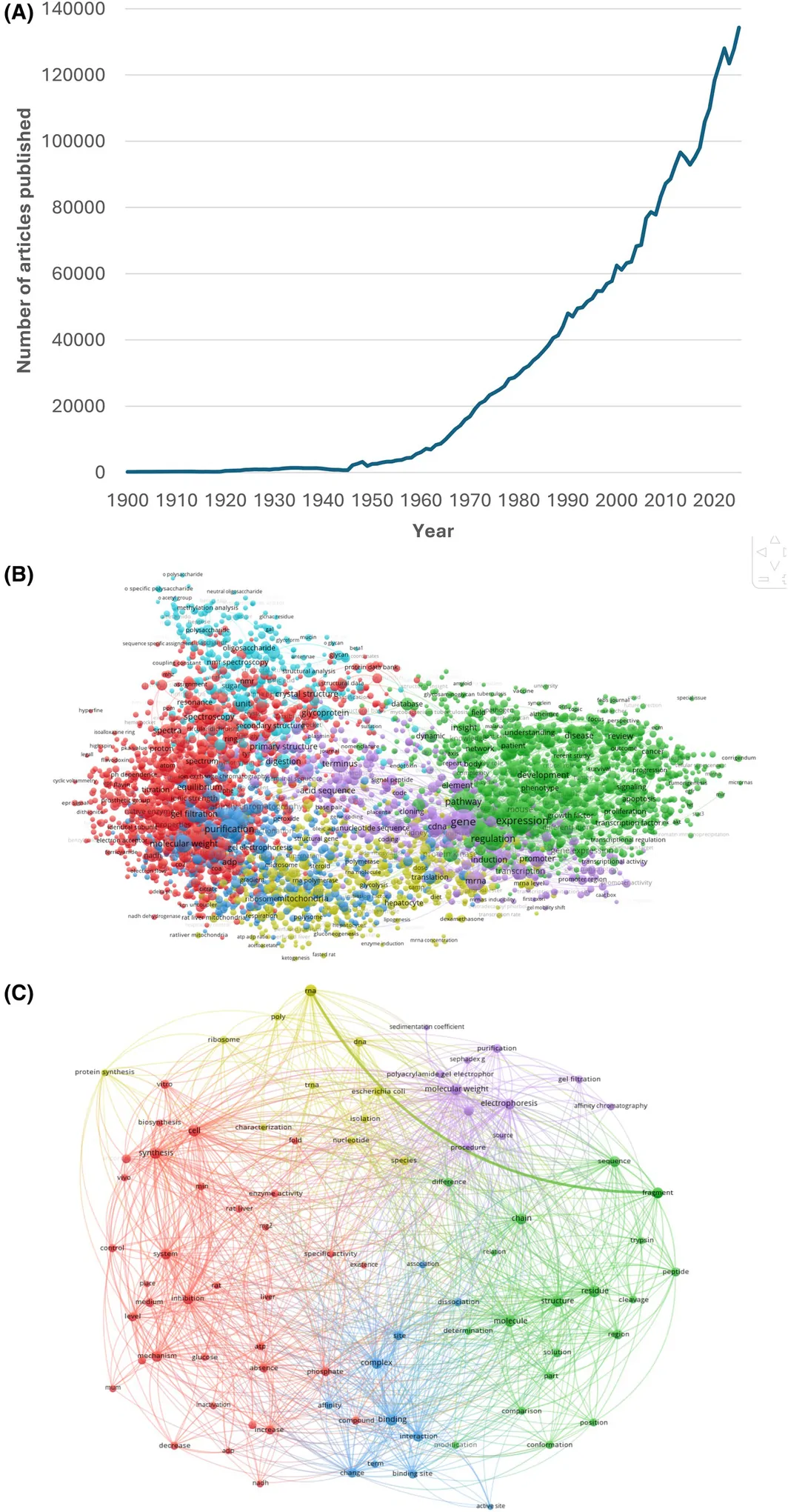
Scientific article number increased and research topics diversified over time. (A) The number of published scientific articles over time in the research area of biochemistry and cell biology [Fields of Research (ANZSRC 2020)]. Data extracted from Dimensions in August 2026. (B) Co‐occurrence word network showing the number and frequency of terms present in the title and abstracts of The FEBS Journal articles published in 2025. Data extracted from Dimensions in August 2026 and visualised using vosviewer. (C) Co‐occurrence word network showing the number and frequency of terms present in the title and abstracts of *The FEBS Journal* articles published in 1975. Data extracted from Dimensions in August 2026 and visualised using vosviewer.

Although external peer review first arose as an editorial necessity, following its standardisation and the establishment of clear ethical guidelines for peer reviewers [4], it has become one of the cornerstones of academic research. The peer‐review process plays a key role in safeguarding the quality, integrity, significance and reproducibility of the scientific record. It benefits not only academic research but also the individuals who take part in it. For authors, the peer‐review process can help improve their manuscript and correct possible errors. For peer reviewers, being part of the process can contribute to their training in critical thinking, help to improve their own work and to keep up with the latest literature, and provide them with a sense of community. Despite the importance of the peer‐review process, there are a limited number of journals with dedicated training programmes for Early Career Researchers (ECRs), and editors have traditionally relied on the input of established academics with the expectation that ECRs will acquire the skills necessary for efficient peer review organically throughout their academic careers.

Since its foundation, one of *FEBS Open Bio's* core values has been to support ECRs. In 2022, we introduced Research Protocols to help combat the reproducibility crisis and highlight the need for validated protocols, particularly when scientists are getting acquainted with a new procedure. With the importance of appropriate training in mind, we are pleased to extend our support to ECRs with the launch of the Early Career Reviewer Hub programme. This initiative is aimed at ECRs (PhD students and postdocs) interested in scientific publishing and who wish to learn about the peer‐review process through actively participating in it. Successful applicants will receive training and guidance from *FEBS Open Bio* editorial staff members and will be involved in the peer review of Research Protocols in their area of expertise. ECRs interested in this initiative are welcome to apply here and to join us for the official project presentation on 22 September, 04:00 PM CET for a webinar hosted in collaboration with the FEBS Junior Section. If you are interested in learning more about this initiative, we invite you to register to the webinar here.

We hope many ECRs will find the Early Career Reviewer Hub a useful learning experience and we can continue to not only support ECRs' professional development but also contribute to the dissemination and promotion of best peer‐review practices for the benefit of the wider scientific community.

## Conflict of interest

The authors declare no conflict of interest.

## Author contributions

SF and MADR wrote the editorial.

## References

[1] 360 Years of Royal Society Publishing The Royal Society. https://royalsociety.org/journals/publishing‐activities/publishing350/publishing360/

[2] Kadar N and Croft RC (2020) Why Semmelweis's doctrine was rejected: evidence from the first publication of his results by Friedrich Wieger, and an editorial commenting on the results. Br J Hist Sci 53, 389–395. 32616108 10.1017/S0007087420000229

[3] Baldwin (2015) Credibility, peer review, and *nature*, 1945–1990. Notes Rec R Soc Lond 69, 337–352. 26495581 10.1098/rsnr.2015.0029 PMC4528400

[4] (2017) COPE Ethical Guidelines for Peer Reviewers Version. doi: 10.24318/cope.2019.1.9

